# Supplementary material for: Long-Term Effectiveness of Physical Exercise-Based Swallowing Interventions for Older Adults with Dementia in a Day-Care Center
Source: Healthcare (Basel). 2023 Apr 28;11(9):1262. doi: 10.3390/healthcare11091262 (PMC10178008; doi:10.3390/healthcare11091262)
Supplement: Supplementary file 1 [file healthcare-11-01262-s001.zip › healthcare-2269609-supplementary.pdf]

:

Table S1 The objectives and instructions for the swallowing-related exercises

Exercises	Objectives of the exercises	Instructions for the exercises<br>General instructions:
Exercises of the head, neck, and shoulder	To warm up and reduce tension in the muscles related to swallowing in the head, neck, and shoulder areas.	• Neck forward stretch: Place your hands on the back of your head and gently pull your head to look down at your stomach, hold the position for 5 seconds, and then relax your head back up. Repeat this set of exercises 5-10 times.<br>• Neck side stretch: Place your right hand on the left side of your head and gently pull your head down towards your right shoulder, hold the position for 5 seconds, and then reverse the motion on the other side. Repeat this set of exercises 5-10 times.<br>• Shoulder shrug: Lift your shoulders up towards your ears, hold for 5 seconds, then relax your shoulders back down. Repeat this set of exercises 5-10 times.<br>• Shoulder roll: Roll your shoulders forward in a circular motion for 5 rotations, then reverse the circular motion backward for 5 rotations.
Oral exercises of the lip and tongue	To maintain or enhance the functional reserve of the muscles required for the transition from chewing to swallowing.	• Lip stretching: Stretch your lips as wide as you can, then relax. Repeat this set of exercises 5-10 times.<br>• Lip puckering: Pucker your lips as if you were going to kiss someone. Hold the position for 5 seconds, then relax. Repeat this set of exercises 5-10 times.<br>• Tongue protrusion: Stick your tongue out as far as you can, then retract it back into your mouth. Repeat this set of exercises 5-10 times.<br>• Tongue side-to-side movement: Move your tongue from side to side inside your mouth. Repeat this set of exercises 5-10 times.
Effortful swallow	To maintain or enhance the functional reserve of the muscles used in swallowing.	• Take a small sip of saliva and hold it in your mouth, then swallow the saliva with as much effort as possible, imagining that you are pushing the liquid or food all the way down to your stomach. After

		you swallow, exhale slowly and relax. Repeat this set of exercises 5 to 10 times.
Vocal function exercise	To maintain or enhance the functional reserve of the muscles involved in vocal fold regulation for swallowing safety.	• Effortful pitch glides: Begin by making a low-pitched "mmm" sound, gradually glide your voice up to reach the top of your range, then slowly glide your voice back down the scale to the starting pitch. Repeat this set of exercises 5 to 10 times.<br>• Sustained hum: Take a deep breath in through your nose, and as you exhale, start to make a humming sound and Keep the hum going for as long as you can. Repeat this set of exercises 5 to 10 times.
Chin tuck against resistance (CTAR)	To strengthen the suprahyoid muscles involved in opening the upper esophageal sphincter at the late stage of swallowing.	• Sustained CTAR (Isometric): Hold a rubber ball under your chin with your hand and keep it in position during the exercise, sustain chin tuck against the ball as hard as possible for 5 seconds, then release and return to the starting position. Repeat this set of exercises 5 times. Rest for 30 seconds between each set of the exercise.<br>• Repetitive CTAR (Isokinetic): Hold a rubber ball under your chin with your hand and keep it in position during the exercise, tuck your chin against the ball as hard as possible and then release it, and repeat the motion 5 times. Repeat this set of exercises 5 times. Rest for 30 seconds between each set of the exercise.
Breathing exercises<br>.	To help improve respiratory function and support swallowing function	• Abdominal breathing: Inhale deeply through your nose, feeling your belly expand as you breathe in, hold your breath for 5 seconds, then exhale slowly through your mouth, feeling your belly contract as you breathe out. Repeat this set of exercises 5 times. As you do this exercise, focus on relaxing your body.
